# Effects of Forest Gaps on the Structure and Diversity of Soil Bacterial Communities in Weeping Cypress Forest Plantations

**DOI:** 10.3389/fmicb.2022.882949

**Published:** 2022-05-16

**Authors:** Qian Lyu, Yan Luo, Yuliang Dong, Yongqi Xiang, Kuangji Zhao, Gang Chen, Yuqin Chen, Chuan Fan, Xianwei Li

**Affiliations:** ^1^College of Forestry, Sichuan Agricultural University, Chengdu, China; ^2^Institute of Forest Genetics and Breeding, Sichuan Academy of Forestry, Chengdu, China; ^3^Key Laboratory of National Forestry and Prairie Bureau on Forest Resources Conservation and Ecological Security in the Upper Reaches of Yangtze River, Sichuan Agricultural University, Chengdu, China; ^4^Forestry Ecological Engineering in Upper Reaches of Yangtze River Key Laboratory of Sichuan Province, Sichuan Agricultural University, Chengdu, China

**Keywords:** forest gaps, forest plantation, soil bacterial community, soil physicochemical properties, indicator bacteria

## Abstract

The decline in forest ecological function caused by pure forest plantations planted in the Yangtze River basin is becoming increasingly serious. To investigate this problem, we selected the local low-efficiency weeping cypress plantations for forest gap transformation. Three forest gap sizes, specifically large, medium, and small gaps, were established, and the effects of gap sizes on soil bacterial community structure and diversity in winter and summer were studied compared to no gaps (CK; control). Compared to CK, forest gaps had a significant effect on soil organic carbon (SOC) and soil total nitrogen (TN), and the highest values of SOC and soil TN under two seasons occurred in large forest gaps. The interactions of forest gap sizes and seasons had significant effects on pH, SOC, TN, and alpha diversity indices, including Simpson, Chao1, and ACE indices. Compared to winter, forest gaps significantly increased the soil bacterial community diversity indices in summer. Forest gap sizes significantly affected the composition of the bacterial community, but the composition of the dominant bacteria at the phyla and genera levels was similar. Linear discriminant effect size (LEfSe) analysis showed that there were 32 indicator bacterial species in two seasons. Co-occurrence network analysis revealed that the relationship of the soil bacterial community at the phyla level was complex, and there was a significant positive correlation among bacterial species. Soil bulk density (BD) and soil moisture (SM) significantly affected the soil bacterial alpha diversity indices. The composition of the dominant bacteria at the phyla level was significantly affected by soil microbial carbon (MBC), whereas the composition of dominant bacteria at the genera level was affected by soil hydrolysable nitrogen (AN) and the carbon/nitrogen (C/N) ratio. In this study, compared to the other forest gaps, large forest gaps were more conducive to the accumulation of soil nutrients, thus improving the structure of the soil bacterial community. Importantly, changes in the soil bacterial community structure due to gap formation may have profound effects on soil biogeochemical processes in weeping cypress forest plantations.

## Introduction

Forests have become important biological communities participating in the global biogeochemical cycle due to their complex structure, and underground microorganisms also have an impact on ecosystem services (Lladó et al., 2018). However, disturbances related to human activities, including deforestation and environmental changes, lead to significant changes in the forest ecosystem and related biogeochemical processes (Li et al., 2019). China has established many timber forests and economic forests in the Yangtze River basin to protect water resources, but the ecosystem service value of these forests is low due to the early mismanagement of forests and the poor growth of many pure forest plantations (Li et al., 2020), indicating the importance of improving forest plantations. In recent decades, the silvicultural practices based on the formation of forest gaps have been increasingly suggested to promote forest plantation regeneration and restoration (Adamic et al., 2017; Lu et al., 2018).

Numerous studies have focused on natural forest gaps. However, with the increasing significance of forest gaps in maintaining the biodiversity and stability of forest ecosystems (Nygaard et al., 2018), artificial forest gaps have been widely adopted in silvicultural practices (Han et al., 2020; Mohler et al., 2021), which has existed in local microsites and ecological conditions (Keram et al., 2019), providing opportunities for plant regeneration and sustainable forest development (Mallik et al., 2014; Perreault et al., 2020). Canopy gaps are created by partial deforestation, and gap size is an important determinant of forest structure (Dobrowolska and Veblen, 2008; Mallik et al., 2014). Because gap sizes provide various net radiation, rainfall, and plant transpiration in the forest (Han et al., 2020), the microclimate change caused by the gap greatly affects the biogeochemical cycling and promotes the aboveground or underground forest communities (Feldmann et al., 2018). Some studies have demonstrated that small forest gaps are beneficial for microbial communities (Yang et al., 2017; Wang X. et al., 2021) and that small forest gaps significantly alter the availability of soil nutrients that are conducive to natural forest regeneration (Muscolo et al., 2007; Xu et al., 2016). However, one study also found that forest gap sizes do not result in long-term effects on the soil microbial community in the temperate northern hardwood forest (Lewandowski et al., 2015). In addition, large forest gaps reduce the soil nutrient availability and enzyme activity in the *Cunninghamia lanceolata* stand (Xu et al., 2016). Thus, the response of soil characteristics to forest gap sizes varies among different forest ecosystems.

The soil bacterial community is a dominant group of soil organisms that participate in nutrient cycling and storage in a terrestrial ecosystem (Gans et al., 2005; Zeng et al., 2016). In general, soil physicochemical properties determine the structure of the bacterial community in forest soil (Xia et al., 2016) and the activity of the bacterial community is mainly affected by the supply of soil carbon and nitrogen, as well the C/N ratio (Zechmeister-Boltenstern et al., 2015). Other soil characteristics also affect the soil bacterial community composition and diversity, such as pH and soil moisture (SM). When soil pH is close to neutral, the soil bacterial community diversity and structural richness reach peak values (Ramirez et al., 2012). SM directly affects the enzyme activity, thus influencing the bacterial community structure (Moorhead and Sinsabaugh, 2000; Li et al., 2021). Although many major factors affecting soil bacterial community have been studied, it is unclear which soil factors play crucial roles in the soil bacterial community structure in forest gaps.

Weeping cypress (*Cupressus funebris*) has been widely used for afforestation in the southern part of China (Li et al., 2019), especially in the Yangtze River basin, which is the third largest river basin in the world, to provide timber or fuelwood and to control erosion in degraded areas due to its rapid growth and adaptation to environmental changes (Lin et al., 2004). The upper reaches of the Yangtze River are an important ecological barrier in China, but the ecological benefits of large-scale weeping cypress plantations are low due to the high initial planting density and mono specificity (Wang Y. et al., 2021). Therefore, the transformation of weeping cypress plantations is particularly important. Previous studies have mainly analyzed the effects of forest gaps on soil physicochemical properties (He et al., 2015), plant diversity (Lyu et al., 2021), and soil microbial community to evaluate the regulation of forest gaps in the forest environment. However, few studies have determined the key forest gap sizes for plantation transformation and the key soil factors that affect the soil bacterial community structure. Therefore, understanding the main factors driving the bacterial community after the formation of forest gaps would help to improve the ecological service function of forest plantation.

In this study, we hypothesized that compared to other forest gap sizes, large forest gaps are more conducive to the accumulation of soil nutrients, thus promoting increases in the bacterial community diversity and improving the bacterial community composition. We employed three sizes of forest gaps and compared to no gaps in weeping cypress plantations to explore the response of the soil bacterial diversity and composition to forest gaps in winter and summer. The main objectives of this study were (1) to explore the response of the bacterial composition and diversity to forest gap sizes, (2) to identify the key soil factors affecting the composition and diversity of the soil bacterial community, and (3) to determine the optimal forest gap size for the reconstruction of weeping cypress plantations.

## Materials and Methods

### Study Site and Sampling

**Figure 1:**
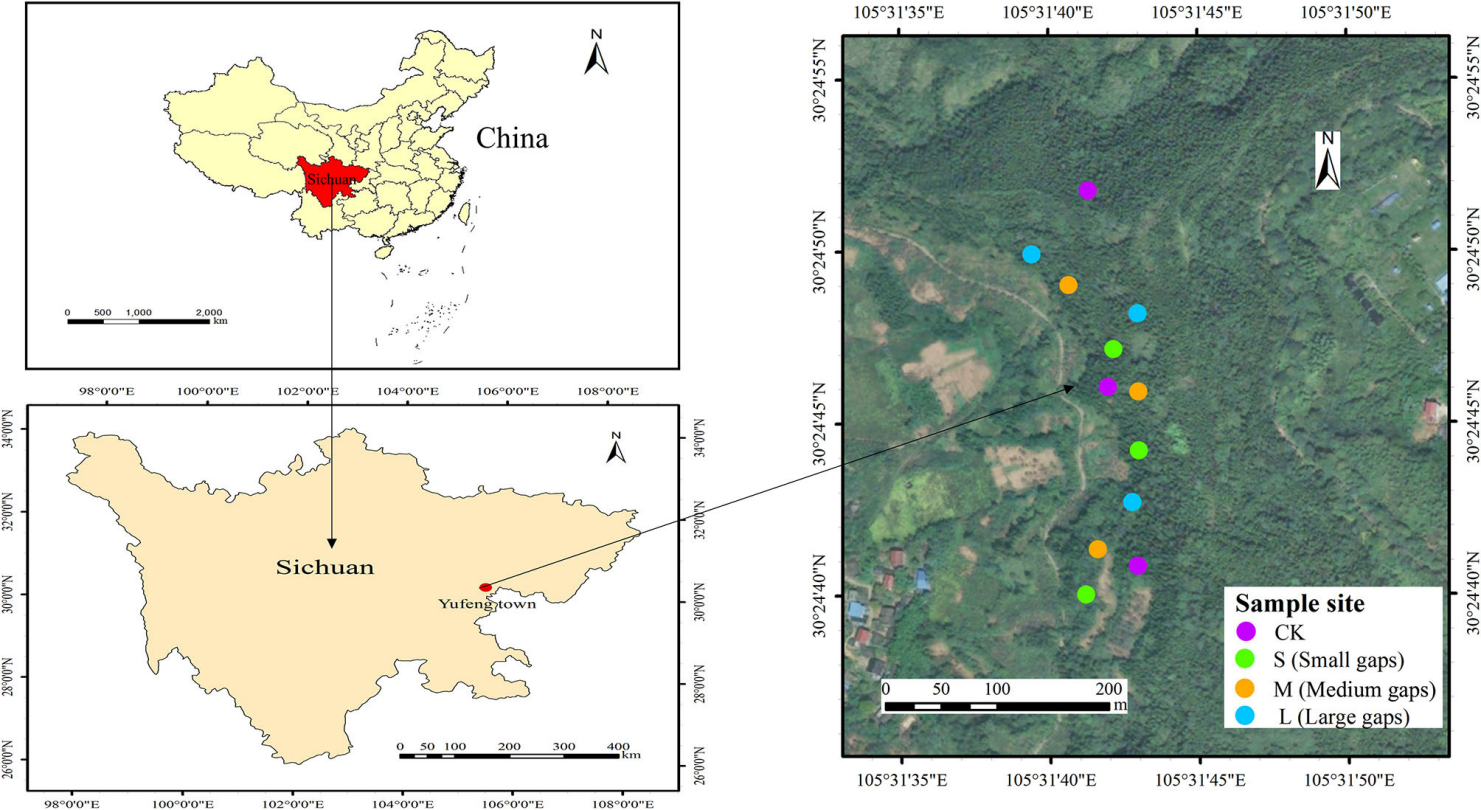
Location of sampling sites in the study area.

The study area was located in Yufeng Town, Suining City, Sichuan Province, China, which belonged to the upper reaches of the Yangtze River basin (30°25′06″ N, 105°32′19″ E). The mean annual temperature is ~17.4°C, and the study area is characterized by a subtropical monsoon climate with a mean annual precipitation of 930 mm. The study site was agricultural land before 1990 until the afforestation project of the long-control protection forest was initiated after 1990 when all pure weeping cypress forests were planted. The soil type is calcareous purple soil, and the dominant understory species include *Coriaria nepalensis, Myrsine africana, Rhus chinensis, Vitex negundo, Ficus tikoua*, and *Stenoloma chusanum*. In October 2015, nine forest gaps with three sizes (Figure 1), namely, small gaps (50–100 m^2^, *n* = 3), medium gaps (100–200 m^2^, *n* = 3), and large gaps (400–667 m^2^, *n* = 3), were created by cutting trees, and the forest gaps were surrounded by closed canopy transition zones and a 5-m buffer with similar elevations and slopes in weeping cypress plantations (Lyu et al., 2021). A laser distance meter (LDM-80H) was used to determine forest gap sizes. For the control (no gaps, CK), non-gap plots with three plot areas of 20 × 20 m were selected in full canopy-covered weeping cypress plantations. All gaps were approximately circular, with west-facing, slopes ranging from 13° to 15°. The mean height of gap border trees was 12.67 m, with mean breast diameter of 10.96 cm (Table 1).

**Table 1:** Basic conditions of the study sites.

**Canopy gap levels**	**Mean height (m)**	**Mean breast diameter (cm)**	**Altitude (m)**	**Slope (α/**°**)**	**Aspect**
CK	11.33 ± 0.58	9.00 ± 1.00	410	14	W
S	12.33 ± 1.53	9.00 ± 1.00	408	13	SW
M	12.33 ± 0.58	11.33 ± 0.58	405	14	W
L	14.67 ± 1.53	14.50 ± 0.50	400	15	W

*S, small gaps; M, medium gaps; L, large gaps; CK, no forest gaps*.

Soil samples were collected in December (winter) of 2019 and July (summer) of 2020, consisting of a mixture of 0–10 cm deep soil in the east, south, west, north, and center points of each plot (Wang Y. et al., 2021). Each soil sample was packed in a sterile bag and then shipped back to the laboratory. Soil samples were sieved through 2-mm mesh and divided into two parts. One subsample was stored in a freezer at −80°C for microbial analysis, and another subsample was utilized to determine soil physicochemical properties.

### Soil Physicochemical Properties Analyses

Soil total nitrogen (TN) was measured using the Kjeldahl method (Kerfahi et al., 2019), and soil hydrolysable nitrogen (AN) was measured by the alkali-hydrolyzed diffusion absorption method. Total phosphorus (TP) was measured using the alkali fusion-Mo-Sb anti-spectrophotometric method, and soil-available phosphorus (AP) was measured by extracting subsamples with 0.03 M NH_4_F– 0.025 M HCl. Soil microbial biomass was measured by chloroform fumigation, and soil microbial carbon (MBC) was determined using a TOC analyzer (Liqui TOC II, Elementar, Germany). Soil microbial nitrogen (MBN) was determined using ultraviolet spectrophotometric colorimetry (Hitachi UV2300; Yan et al., 2020). For pH analysis, a 10-g soil sample was added to a small jar with 25 ml of CaCl_2_ solution, and the sample was shaken for 30 min followed by measurement of the pH using a pH meter. Soil organic carbon (SOC) was determined by hydration with the potassium dichromate oxidation-colorimetric method. Soil samples were weighed, oven-dried at 105°C, and weighed again to determine SM. Soil bulk density (BD) was obtained by dividing the dry soil mass by the (known) volume of the sampling core and expressed as g/cm3 (Mora and Lázaro, 2014).

### DNA Extraction, Amplification, and Sequencing

DNA extraction, bacterial 16S rRNA gene amplification, and sequencing were performed as previously described (Shen et al., 2013; Burns et al., 2016). Microbial DNA was extracted using the HiPure Soil DNA extraction Kit (Magen, Guangzhou, China). The PCR amplification conditions of the V3–V4 region of the 16s rRNA gene were 94°C for 2 min; 30 cycles of 98°C for 10 s, 62°C for 30 s and 68°C for 30 s; and 68°C for 5 min. The V3–V4 highly variable regions of the bacterial 16S rRNA genes were amplified by Illumina sequencing (Illumina NovaSeq 6000) using the following primers, namely, 341F, CCTACGGGNGGCWGCAG; and 806R, GGACTACHVGGGTATCTAAT. The PCR assay was performed in triplicate. The amplification system (a total of 50 μl) included 5 μl of 10 × KOD buffer, 5 μl of 2 mM dNTPs, 3 μl of 25 mM MgSO_4_, 1.5 μl of upstream and downstream primers (10 μM), 1 μl of KOD polymerase, and 100 ng of template DNA. The amplified fragments were separated on a 2% agarose gel, extracted, and purified using AxyPrep DNA gel extraction kits (Axygen Biosciences, Union City, CA, USA) according to the instructions of the manufacturer, and the fragments were quantified using ABI StepOnePlus real-time PCR systems (Life Technologies, Foster City, USA). According to the standard operation, the purified fragments were sequenced by double-terminal sequencing (PE250) on the Illumina platform.

### Bioinformatics Analysis

The raw data of the Illumina platform were filtered using FASTP (version 0.18.0; Guo et al., 2017), and the filtered clean reads were used for assembly analysis. FLASH (version 1.2.11) was used to merge clean reads into a tag with a threshold of minimum overlap of 10 bp and a maximum mismatch rate of 2% (Magoč and Salzberg, 2011). QIIME (version 1.9.1) was used to filter low-quality tags to obtain high-quality clean tags (Ramírez-Guzmán et al., 2004). Based on the reference database (version r20110519, http://drive5.com/uchime/uchime_download.html), the UCHIME algorithm was used to check the chimera of the tag, and the clean tag obtained from the chimera was filtered for follow-up analysis (Edgar et al., 2011). Using the UPARS (version 9.2.64) process, a clean tag was clustered into an operational taxon using operational taxonomic units (OTUs) according to ≥97% similarity (Edgar et al., 2011). The tag sequence with the highest abundance was selected as the representative sequence of each OTU. We uploaded all raw sequences to the National Center for Biotechnology Information Sequence Read Archive under submission number SUB10947982 and BioProject number PRJNA796764 for data analysis.

### Data Analysis

One-way analysis of variance (ANOVA) was performed using SPSS version 20.0 (SPSS, Chicago, IL, USA) to analyze the significant differences in the soil physicochemical properties and soil bacterial community structure among forest gaps in winter and summer. Two-way ANOVA was analyzed to test whether there was a significant interaction among the forest gap sizes, seasons, soil bacterial alpha diversity, and soil physicochemical properties. Principal coordinate analysis (PCoA) and permutational multivariate analysis of variance based on the Bray-Curtis distance were conducted in R 3.4 software (http://www.r-project.org/) to evaluate the general changes in the soil bacterial community between gaps. A nonparametric factor Kruskal-Wallis (KW) sum-rank test was applied to identify significant taxa, and linear discriminant analysis (LDA) was applied to evaluate the effect of each feature. An LDA threshold of 3.0 and a significant *p*-value were used to detect biomarkers. An absolute value of the correlation coefficient >0.5 and *p* < 0.05 were considered as the threshold for screening. After displaying the results that met the conditions, we selected the top 50 correlation pairs and generated the co-occurrence network of the bacterial community at the phyla and species levels. CANOCO 5.0 was used to analyze the bacterial diversity, dominant species, and soil physicochemical properties under gap sizes by redundancy analysis (RDA).

## Results

### Variations in Soil Physicochemical Properties

In winter, compared to CK, forest gaps significantly affected the SOC, TN, and the C/N ratio (*p* < 0.05; Table 2). Interestingly, both SOC and TN were highest in large gaps. The soil pH values ranged from 7.52 to 7.86, and the soil pH values were only significantly different in the large and small forest gaps compared to CK (*p* < 0.05). Compared to CK, the SM increased by 16.54 and 37.65% in the medium and large forest gaps, respectively (*p* < 0.05). In summer, compared to CK, there were significant differences in TN among the forest gap sizes (*p* < 0.05; Table 2) with increases of 2.86 and 17.14% in the medium and large forest gaps, respectively, but a decrease of 18.10% in small forest gaps. Both SOC and the C/N ratio of small forest gaps were significantly lower than those of CK (*p* < 0.05). The seasons significantly affected SOC (*p* < 0.001), TN (*p* < 0.001), TP (*p* < 0.001), MBC (*p* < 0.001), MBN (*p* < 0.001), and AN (*p* < 0.05). The interactions of forest gap sizes and seasons had significant effects on pH, SOC, and TN (Supplementary Table 1, *p* < 0.001).

**Table 2:** Soil physicochemical properties among forest gap sizes.

**Canopy gap levels**	**pH**	**SOC (g/kg)**	**TN (g/kg)**	**C/N**	**TP (g/kg)**	**AP (mg/kg)**	**AN (mg/kg)**	**MBC (mg/kg)**	**MBN (mg/kg)**	**BD (p/g cm-3)**	**SM (w/%)**
D-S	7.86 ± 0.01a	12.63 ± 1.05d	1.03 ± 0.03d	12.25 ± 1.01b	0.84 ± 0.02a	10.34 ± 1.64a	86.59 ± 13.35b	24.49 ± 9.61a	5.03 ± 0.77a	1.46 ± 0.11a	24.33 ± 2.98c
D-M	7.66 ± 0.05b	15.88 ± 1.05c	1.29 ± 0.05b	12.33 ± 0.87b	0.62 ± 0.01b	7.44 ± 0.78b	144.78 ± 75.32ab	23.21 ± 3.99a	3.66 ± 2.01a	1.34 ± 0.02ab	28.82 ± 0.72b
D-L	7.52 ± 0.10c	19.34 ± 0.16a	1.59 ± 0.03a	12.17 ± 0.35b	0.64 ± 0.02b	6.62 ± 0.72b	219.73 ± 90.73a	21.87 ± 2.95a	4.46 ± 1.26a	1.08 ± 0.05c	34.04 ± 1.66a
D-CK	7.67 ± 0.03b	17.54 ± 0.54b	1.19 ± 0.04c	14.76 ± 0.78a	0.66 ± 0.03b	7.68 ± 1.87b	83.97 ± 8.36b	14.30 ± 7.53a	3.41 ± 1.34a	1.24 ± 0.15bc	24.73 ± 0.58c
S-S	7.67 ± 0.15a	9.58 ± 1.26c	0.86 ± 0.03c	11.22 ± 1.80b	0.77 ± 0.06a	7.52 ± 0.32a	61.64 ± 12.57a	48.29 ± 8.16a	0.52 ± 0.17a	1.34 ± 0.08a	24.96 ± 0.47a
S-M	7.81 ± 0.04a	13.72 ± 0.35b	1.08 ± 0.03b	12.74 ± 0.53ab	0.56 ± 0.06b	6.58 ± 1.29a	99.11 ± 24.13a	56.94 ± 10.32a	1.79 ± 0.86a	1.34 ± 0.13a	30.15 ± 3.64a
S-L	7.79 ± 0.05a	17.11 ± 1.31a	1.23 ± 0.02a	13.88 ± 1.16a	0.54 ± 0.03b	6.88 ± 0.24a	101.50 ± 18.11a	59.58 ± 13.15a	1.16 ± 0.72a	1.24 ± 0.14a	33.39 ± 7.53a
S-CK	7.68 ± 0.12a	15.82 ± 0.84a	1.05 ± 0.01b	15.03 ± 0.85a	0.55 ± 0.07b	7.64 ± 1.37a	87.02 ± 25.40a	62.10 ± 39.42a	1.11 ± 0.99a	1.21 ± 0.06a	31.48 ± 3.05a

*D-S, small gaps in winter; D-M, medium gaps in winter; D-L, large gaps in winter; D-CK, no forest gaps in winter. S-S, small gaps in summer; S-M, medium gaps in summer; S-L, large gaps in summer; S-CK, no forest gaps in summer*.

*Data are expressed as the average ± SD*.

*Different letters in the same column denote statistically significant at p < 0.05, based on a one-way ANOVA followed by an LSD test*.

### Soil Bacterial Community Diversity of Forest Gap Sizes

**Figure 2:**
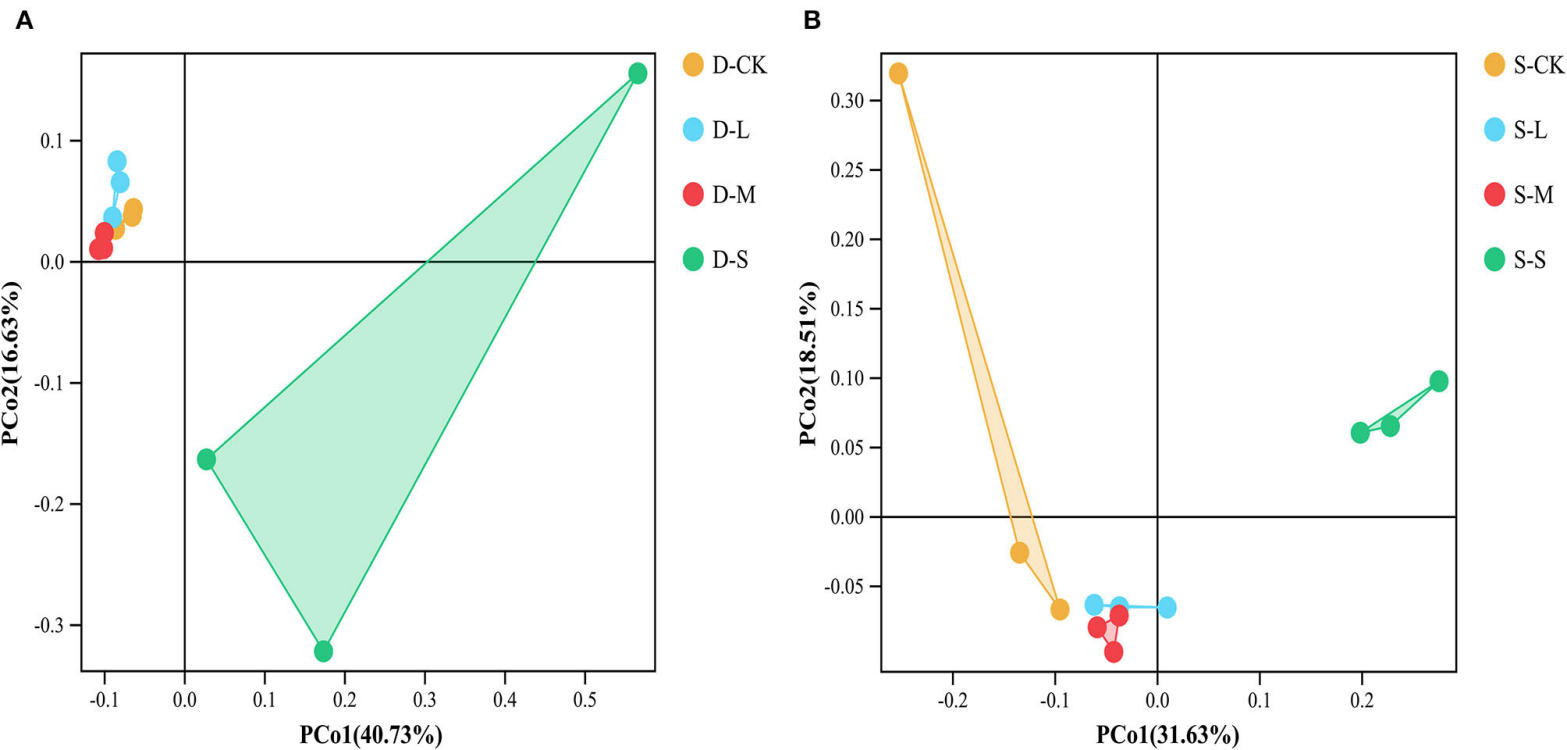
Principal coordinates analysis (PCoA) of the bacterial community composition based on the Bray–Curtis distance among different groups in **(A)** winter and **(B)** summer. D-S, small gaps in winter; D-M, medium gaps in winter; D-L, large gaps in winter; D-CK, no forest gaps in winter; S-S, small gaps in summer; S-M, medium gaps in summer; S-L, large gaps in summer; S-CK, no forest gaps in summer.

In winter, compared to CK, the small forest gaps significantly reduced Chao1 and ACE index values (*p* < 0.05; Table 3). In summer, compared to CK, there were significant differences in the soil bacterial alpha diversity as indicated by Shannon, Simpson, Chao1, and ACE index values among the forest gap sizes (*p* < 0.05; Table 3). Shannon and Simpson index values were the highest in the small forest gaps, but the Chao1 and ACE index values were the highest in the large forest gaps. The seasons significantly affected the Simpson and Chao1 index values (*p* < 0.05), and the interactions of forest gap sizes and seasons had significant effects on the Simpson, Chao1, and ACE (Supplementary Table 1, *p* < 0.05). PCoA (Figure 2A) and analysis of similarities (ANOSIM) (Supplementary Figure 1A) of the soil bacterial communities showed significant variations in winter (*R* = 0.568, *p* = 0.001) among forest gap sizes. PCoA of the soil bacterial communities among the forest gap sizes showed significant changes (Figure 2B). The ANOSIM tests indicated that the soil bacterial communities were significantly different in summer (*R* = 0.614, *p* = 0.001; Supplementary Figure 1B).

**Table 3:** Changes in soil bacterial alpha diversity among forest gap sizes.

**Canopy gap levels**	**Shannon**	**Simpson**	**Chao1**	**ACE**
D-S	9.61 ± 0.17a	0.995 ± 0.0010a	4875.74 ± 680.30b	4891.21 ± 683.07b
D-M	9.31 ± 0.25a	0.991 ± 0.0026b	6182.25 ± 438.50a	6267.80 ± 527.17a
D-L	9.43 ± 0.01a	0.992 ± 0.0008b	5985.02 ± 69.64a	6000.80 ± 75.93a
D-CK	9.49 ± 0.09a	0.994 ± 0.0009ab	6156.17 ± 164.78a	6276.72 ± 199.28a
S-S	9.53 ± 0.28a	0.9951 ± 0.0014a	4562.50 ± 303.39a	4884.46 ± 310.45a
S-M	9.47 ± 0.09a	0.9943 ± 0.0008a	4578.88 ± 43.21a	4923.87 ± 44.16a
S-L	9.49 ± 0.18a	0.9946 ± 0.0011a	4683.32 ± 182.33a	5006.28 ± 168.36a
S-CK	8.94 ± 0.36b	0.989 ± 0.0022b	4163.70 ± 190.91b	4442.94 ± 154.66b

*D-S, small gaps in winter; D-M, medium gaps in winter; D-L, large gaps in winter; D-CK, no forest gaps in winter; S-S, small gaps in summer; S-M, medium gaps in summer; S-L, large gaps in summer; S-CK, no forest gaps in summer*.

*Data are expressed as the average ± SD*.

*Different letters in the same column denote statistically significant at p < 0.05, based on a one-way ANOVA followed by an LSD test*.

### Soil Bacterial Community Distribution and Composition of Forest Gap Sizes

**Figure 3:**
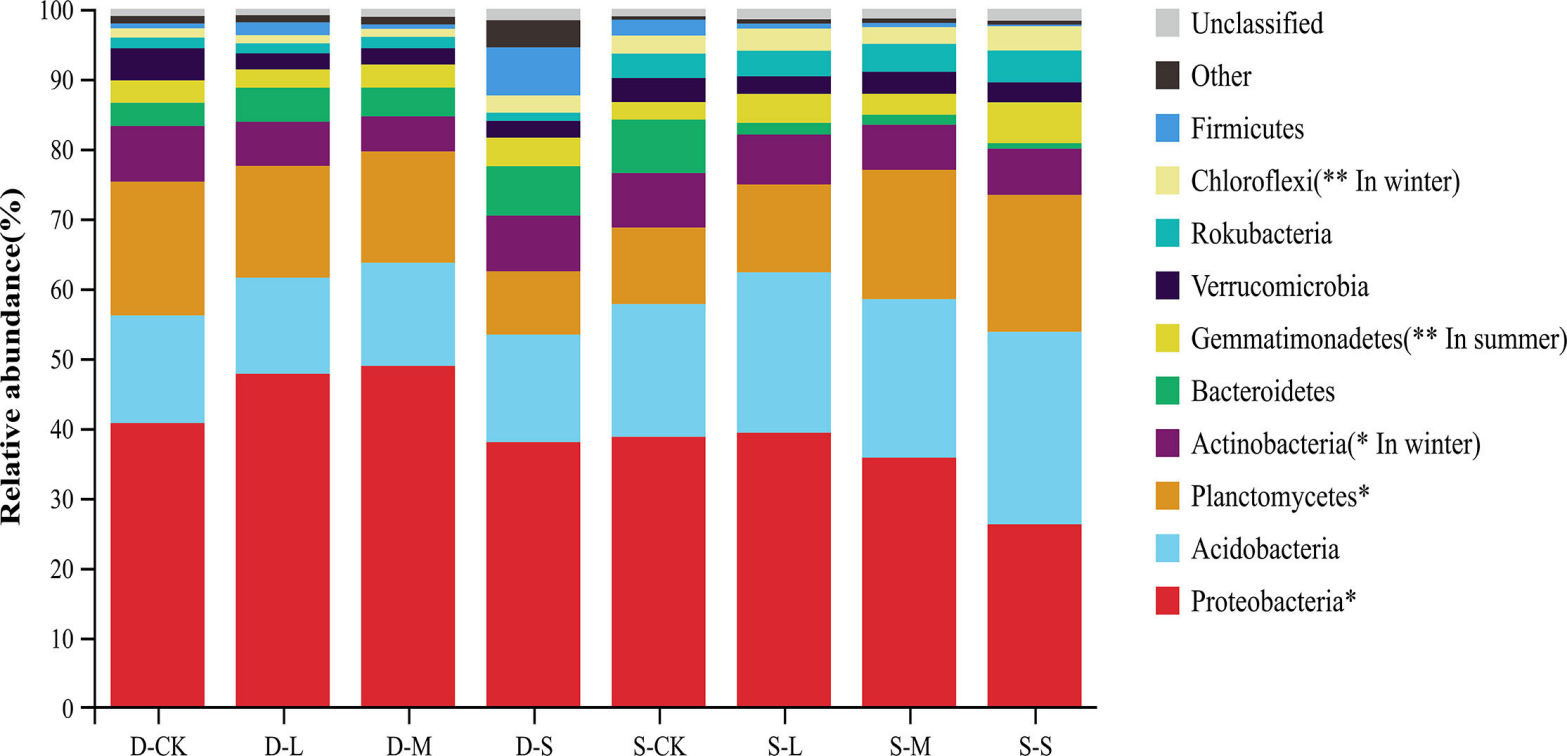
Relative abundance of bacteria taxa at the phyla level. Only the taxa with the average relative abundance of the top 10 are shown. *p < 0.05; **p < 0.01. D-S, small gaps in winter; D-M, medium gaps in winter; D-L, large gaps in winter; D-CK, no forest gaps in winter; S-S, small gaps in summer; S-M, medium gaps in summer; S-L, large gaps in summer; and S-CK, no forest gaps in summer.

At the phyla level, Proteobacteria were considered predominant in all treatments. The relative abundances of Proteobacteria and Planctomycetes significantly differed across all treatments at the phyla level. However, the relative abundances of Proteobacteria and Planctomycetes showed an opposite trend in the two seasons. The average relative abundance of Proteobacteria in winter was higher than that in summer, whereas the average relative abundance of Planctomycetes in winter was lower than that in summer. The relative abundance of Actinobacteria with forest gaps was significantly decreased in winter compared to CK (*p* < 0.05). However, the relative abundance of Chloroflexi with forest gaps significantly increased by 1.89, 0.87, and 0.91 times in the small, medium, and large forest gaps, respectively, in winter (*p* < 0.01). In summer, compared to CK, the relative abundance of Gemmatimonadetes increased by 133.20, 19.20, and 64.80% in the small, medium, and large forest gaps, respectively (Figure 3, *p* < 0.01).

In winter, significant differences in the relative abundances of the genera *Dongia, Sphingomonas, Gemmata*, and *Steroidobacter* were found among samples (*p* < 0.05). Compared to CK, the relative abundances of *Dongia, Sphingomonas*, and *Steroidobacter* were significantly higher in the large and medium forest gaps, but significantly lower in the small forest gaps. The relative abundance of *Gemmata* significantly decreased with the decrease in gap size and was lower than CK. In summer, the forest gap sizes only had a significant effect on the relative abundance of RB41 (Supplementary Figure 2, *p* < 0.01).

**Figure 4:**
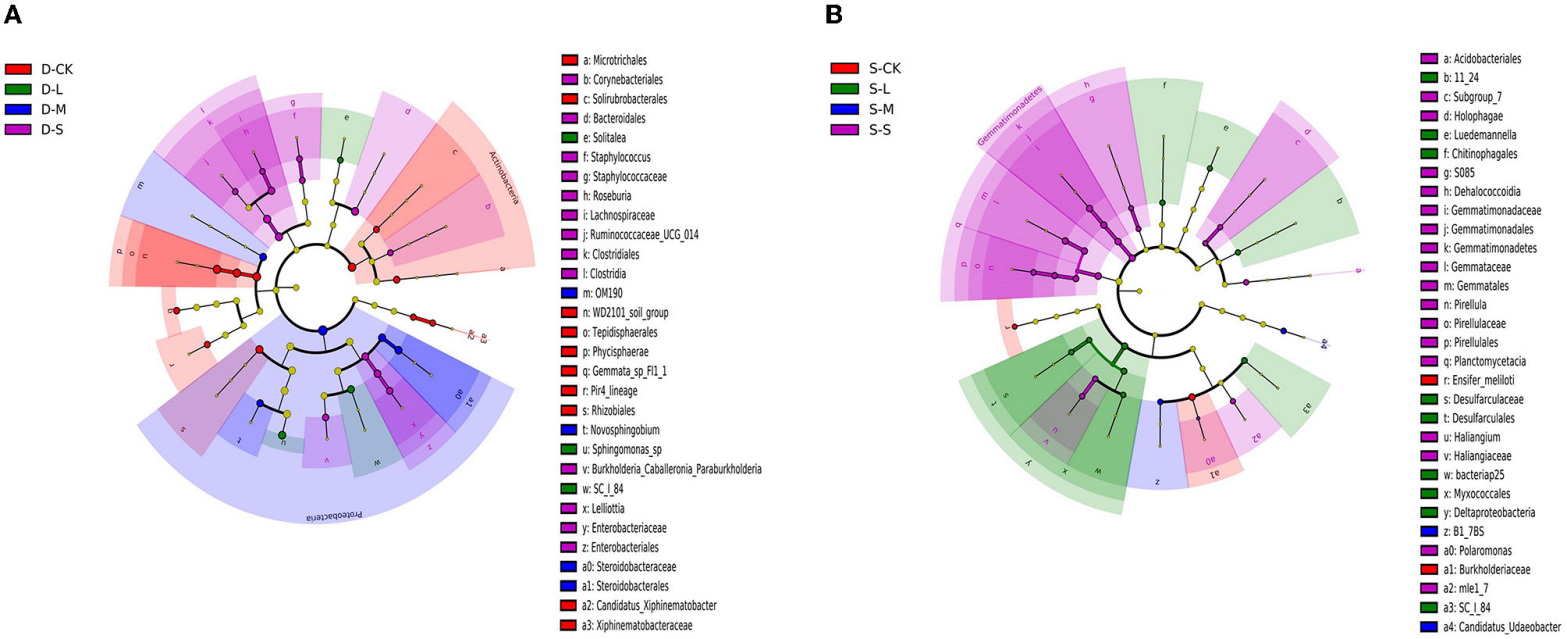
Bacterial linear discriminant effect size (LEfSe) analysis of forest soil among forest gap sizes. Cluster tree of bacterial LEfSe analysis in **(A)** winter and **(B)** summer. Circle radiation from inner to outer of evolutionary branch figure represents the classification of the level from the phylum to species. Each small circle represents the level of a classification in different classification levels. The diameter of the circle is proportional to the relative abundance. The species without significant differences uniformly color to yellow, and the other species are colored according to the highest abundance of the species. The taxa represent the kingdom, phylum, class, order, family, and genus levels from the center outward. Only taxa meeting an LDA significance threshold of >3 are shown. D-S, small gaps in winter; D-M, medium gaps in winter; D-L, large gaps in winter; D-CK, no forest gaps in winter; S-S, small gaps in summer; S-M, medium gaps in summer; S-L, large gaps in summer; S-CK, no forest gaps in summer.

**Figure 5:**
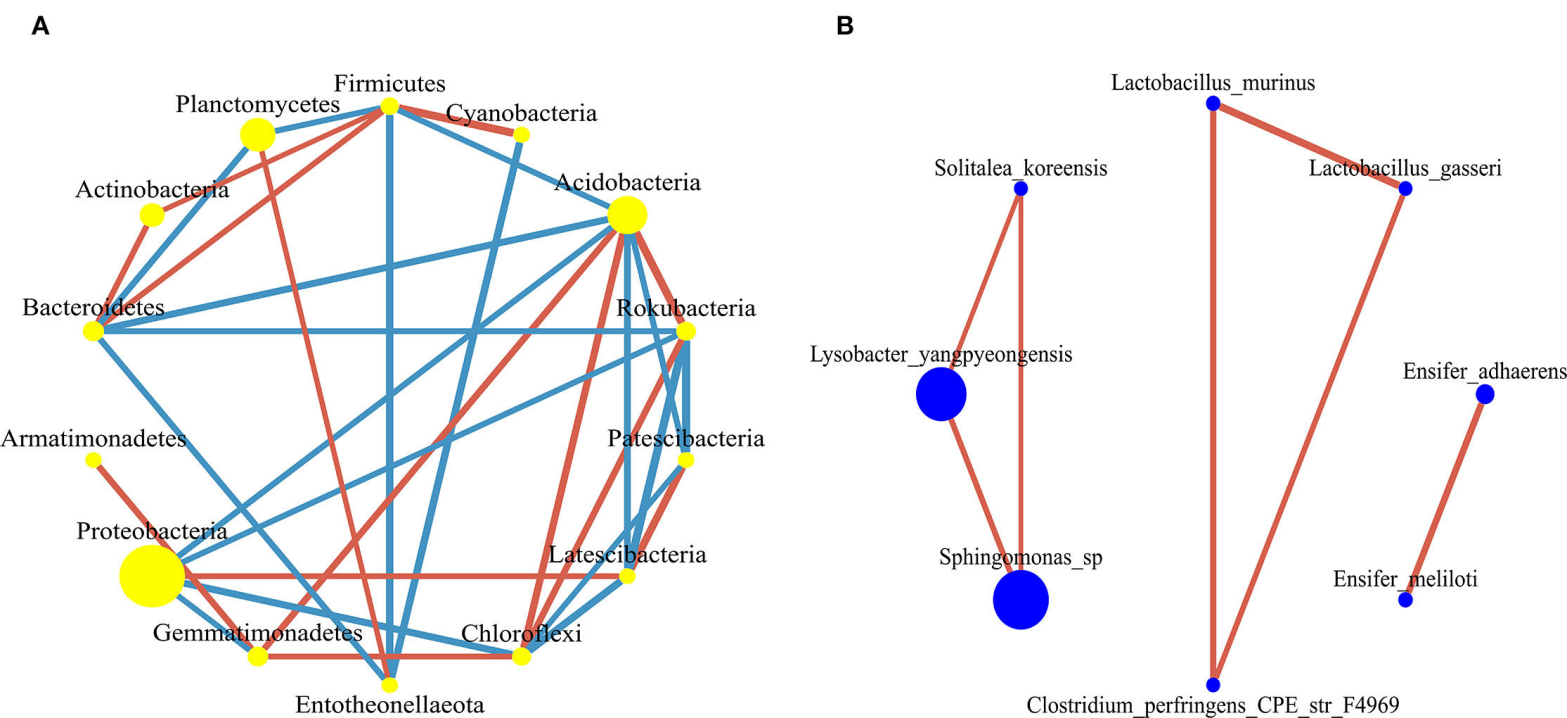
The co-occurrence network of bacterial communities in forest gap sizes at the **(A)** phyla level and **(B)** species level. The nodes in the network represent 14 bacterial phyla and 8 bacterial species. The size of each node is proportional to the relative abundance. The red edge represents the positive correlation between bacteria, and the blue edge represents the negative correlation between bacteria.

There were 32 significantly different biomarkers with LDA effect size scores > 3 in summer and winter (Supplementary Figure 3). Overall, Proteobacteria contributed the most to the weeping cypress forest in winter (Figure 4A and Supplementary Figure 3A) and the largest contribution to the weeping cypress forest in summer was from Burkholderiaceae, followed by Planctomycetacia and Gemmatimonadales (Figure 4B and Supplementary Figure 3B). There were more negative (58.06%) than positive (41.94%) correlations among the 14 phyla (Figure 5A, *p* < 0.05). Several taxa played central roles (high degree of connectivity) in the networks with high relative abundances, such as Proteobacteria and Acidobacteria, indicating that they were the dominant phyla in the bacterial communities. At the species level, there was a significant positive correlation among all species (Figure 5B, *p* < 0.05). In addition, several taxa played central roles (high degree of connectivity) in the networks but had low relative abundances, such as *Lactobacillus_murinus, Clostridium_perfringens_CPE_str_F4969, Lactobacillus_gasseri*, and *Solitalea_koreensis*. Although these taxa had the same connectivity as *Lysobacter_yangpyeongensis* and *Sphingomonas*_sp, they were not the dominant species of the soil bacterial community.

### Relationship Between the Bacterial Community Structure and Soil Properties

**Figure 6:**
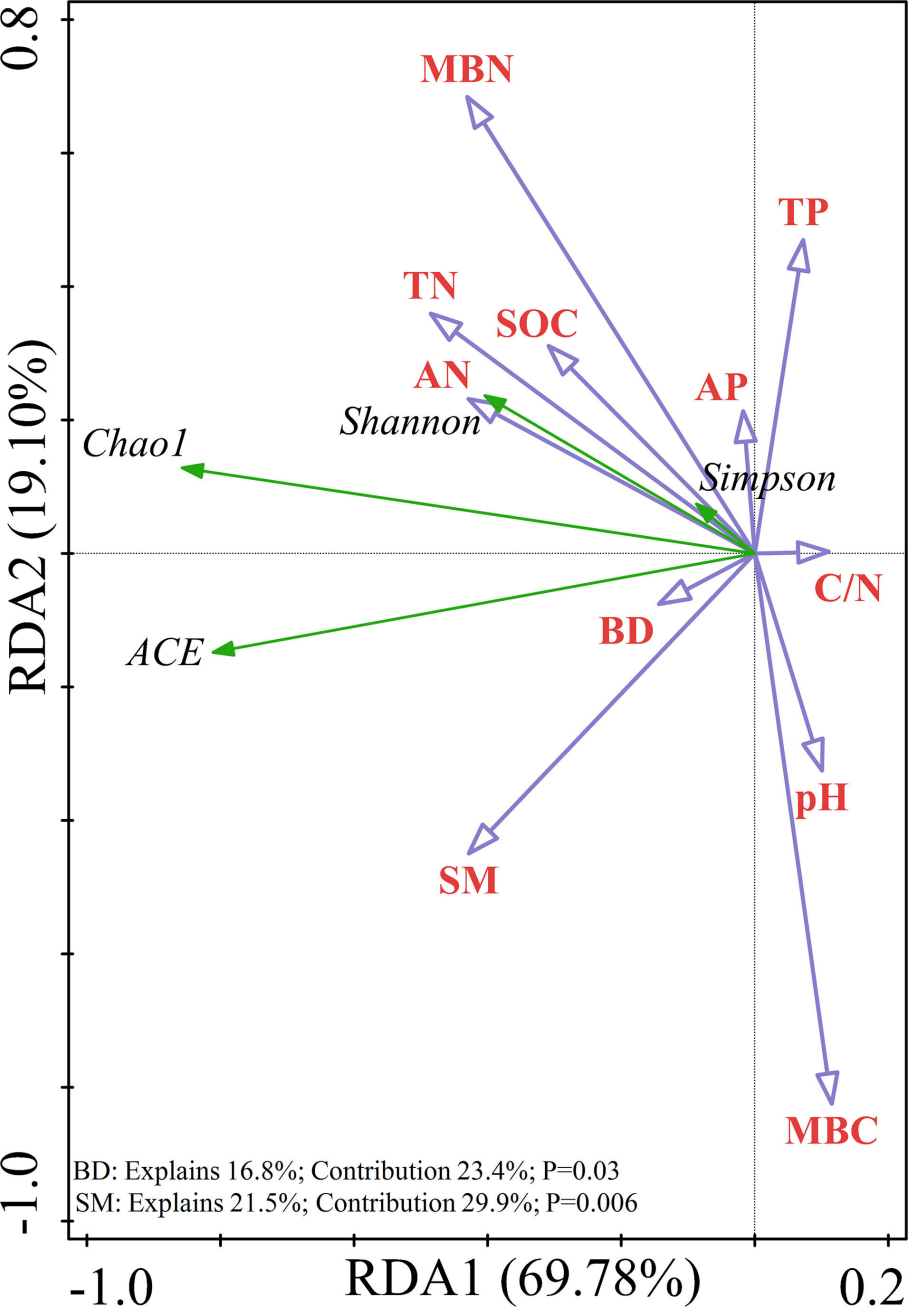
Redundancy analysis (RDA) showing the relationship between soil variables and soil bacterial alpha diversity.

**Figure 7:**
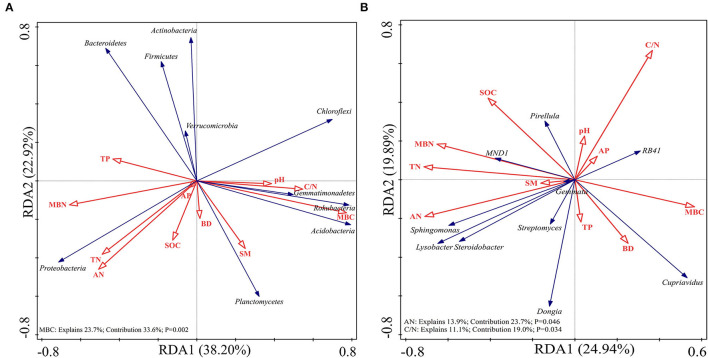
Redundancy analysis (RDA) of soil-dominant bacteria phyla **(A)** and soil-dominant bacteria genus **(B)** constrained by soil variables.

Redundancy analysis was performed on the soil samples to determine which soil physicochemical properties affected the bacterial diversity and composition of the major bacterial communities. All of the edaphic variables explained 88.88% of the variance, with axis 1 explaining 69.78% of the variance and axis 2 explaining another 19.10% of the variance. Combined with the results of the Monte Carlo permutation test, with the exception that the BD (*p* = 0.03) and SM (*p* = 0.006) had a significant effect on bacterial diversity, the contribution of the edaphic variables to bacterial diversity reached 53.3%. All diversity indices were positively correlated with SM and BD (Figure 6). The first and second axes of the RDA explained 38.20 and 22.92% of the variance in the bacterial community composition at the phyla level, respectively. The most important soil property determining the dominant phyla was MBC (*p* = 0.002), which was negatively correlated with the relative abundances of Proteobacteria and Actinobacteria but positively correlated with the relative abundances of Planctomycetes and Acidobacteria (Figure 7A). Figure 7B shows that the total variation was 44.83% in terms of the RDA of the relative abundance of the soil-dominant bacteria genera and soil properties. The dominant bacteria genera were more closely associated with AN (*p* = 0.0046) and the C/N ratio (*p* = 0.0034). For example, AN was negatively correlated with the relative abundances of *RB41* and *Cupriavidus*, but positively correlated with other dominant bacterial genera. The C/N ratio was positively correlated with *RB41* and *Pirellula* but negatively correlated with other dominant bacterial genera.

## Discussion

### Effects of Forest Gaps on Soil Properties

The gap partitioning hypothesis (GPH) suggests that gaps cause heterogeneity of resources, which not only directly changes the physical and chemical properties of woodland soil but also plays an important role in tree regeneration of forests with different canopy sizes (Ricklefs, 1977; Kern et al., 2013). Forest gaps are formed by artificial thinning, which directly induces shifts in litter input (Lin et al., 2015). The input of litter enhances SOC dynamics by increasing soil organic matter input and decreasing the decomposition rate (Cheng and An, 2015; Nadal-Romero et al., 2016). A larger canopy gap accommodates more litter input and accelerates litter decomposition (Yang et al., 2017). In this study, SOC was the highest in both winter and summer under large forest gaps, but SOC was the lowest in small forest gaps. Compared to CK, the effect of forest gaps on TN was significantly higher with large forest gaps, but significantly lower with small forest gaps. Scharenbroch and Bockheim (2007) reported that there is greater nutrient mineralization and leaching in forest gaps than in closed forests, which was consistent with parts of our results. Shortly after artificial thinning, the TN contents decrease due to soil bareness, which strongly increases surface runoff and accelerates erosion (Guillaume et al., 2015), and soil management measures overturn the soil layer, destroy aggregates, and promote the leaching of mineral nitrogen (Sheng et al., 2015), which is one of the main reasons for the decrease in TN in plantations (Maranguit et al., 2017). Due to the low SOC and TN in the small forest gaps, there was also a low C/N ratio. Seasonality is always related to the change in climate and plant growth, inducing variations in soil properties (Shen et al., 2021). Meanwhile, there are certain differences in the forest structure and microenvironmental conditions by forest gaps, leading to differences in soil physicochemical properties (Lyu et al., 2021). Therefore, gap sizes and seasons had a significant impact on pH and SOC and TN. There is growing evidence that canopy openings caused by forest gaps allow more light to reach the surface, resulting in higher temperatures and higher water inputs on the ground (Ni et al., 2016). Moreover, there is lower light radiation intensity in winter than in summer, resulting in an increase of SM in winter under the medium and large forest gaps. Compared to CK, the TP in the small forest gaps was significantly higher, which agreed with the conclusion drawn by Wang X. et al. (2021) that the small forest gaps are the most conducive to the accumulation of soil total nutrients. In summary, regardless of season, the soil SOC, TN, and AN, as well as SM of the large forest gaps, were significantly increased compared to the other forest gap sizes and CK.

### Effects of Forest Gaps on Soil Bacterial Community

The soil bacterial alpha diversity in the forest gaps under weeping cypress forest in summer was more significant than that in winter, which agreed with the findings in a coniferous forest reported by Lin et al. (2018). In general, low temperature reduces microbial diversity in winter and even kills some cold-tolerant bacterial species (Zhao et al., 2016). In the study site of this study, the precipitation is mainly concentrated from June to August, and the average temperature in summer is nearly 20°C higher than that in winter. Therefore, higher temperature and precipitation promote microbial activity and increase soil bacterial diversity in summer (Romanowicz et al., 2016). In addition, plants enter the peak photosynthetic season in summer, and photosynthate entering the soil through the roots also promotes the growth and reproduction of microorganisms (Buckeridge et al., 2013). Forest gaps alter the hydrological and thermal conditions by redistributing effective light and precipitation (Zhang and Zak, 1995), ultimately influencing the distribution and activity of the soil biota (Huang et al., 2020). Seasonal temperature changes also quickly affect the alpha diversity of bacterial community structure (Stark et al., 2007), so significant interactions could be observed between forest gap sizes and seasons. Previous studies on different forest ecosystems have also shown that the disturbance of forest soil increases the diversity of microbial communities (Kaiser et al., 2016; Brödlin et al., 2019). Although the loss of canopy leads to the change in the soil environment in the forest gaps and destroys the balance of the microecosystem, it provides an alternative environment for the distribution and metabolism of different bacteria, thus increasing bacterial diversity (Zhang et al., 2018). A small gap is the initial stage of gap opening (Yang et al., 2017), in which the Simpson index of bacteria should be high because the disturbance is recent (Wang X. et al., 2021), which was confirmed by this study. In forest gaps, the bacterial Chao1 and ACE indices gradually increased with increasing gap size and reached a maximum in the large gaps, which may have been due to the large gaps negatively affecting litter decay (Beckage et al., 2000; Sariyildiz, 2008). In general, the soil bacterial alpha diversity was significantly increased by forest gaps in summer, and the Chao1 and ACE indices of the large forest gaps reached their maximum in summer.

In this study, the soil bacterial communities showed significant variations among the forest gap sizes. The dominant soil bacterial community compositions at the phyla level and genera level were similar. Proteobacteria, Acidobacteria, and Planctomycetes were the predominant bacterial phyla in winter and summer in the cypress forests. Proteobacteria and Acidobacteria, which are acidophilic bacteria, were predominant in the neutral soil of this study, confirming that members of Proteobacteria and Acidobacteria are common in almost all soil types (Zhang and Xu, 2008). This phenomenon is related to the genomic characteristics of ATP-binding cassette transporters that encode affinity (Ward et al., 2009). Some studies have shown that the relative abundance of Actinobacteria significantly increases under high organic carbon and alkaline pH soil conditions (Li et al., 2016; Lin et al., 2018), indicating that the relative abundance of Actinobacteria decreases due to neutral pH and decreased organic carbon content in small- and medium-sized gaps. LEfSe analysis showed that Proteobacteria had the highest contribution to the soil bacterial community in winter, which was related to the high hemoglobin content in the topsoil layer. Proteobacteria use hydropyrite as an electron acceptor to drive iron ore reduction under an anoxic environment and reduce iron-containing organic matter to ferrous atoms, resulting in an increase in heme content (Wang et al., 2020). The relative abundance of Proteobacteria significantly increased in winter and significantly decreased in summer, while the relative abundance of Planctomycetes showed the opposite trend. These findings are supported by the copiotrophic hypothesis (Fierer et al., 2012), which suggests that copiotrophic groups (such as Proteobacteria) with rapid growth rates are more likely to increase under nutrient-rich conditions, while oligotrophic groups (such as Planctomycetes) with slower growth rates may decrease. In this study, the soil properties in winter were higher than those in summer, and the higher properties in winter promoted significant differences in the bacterial genera *Steroidobacter, Sphingomonas*, and *Dongia*, which belong to Proteobacteria. These bacteria are involved in soil denitrification (Marušincová et al., 2013; Asaf et al., 2020) and plant growth promotion (Mukhtar et al., 2018). The relative abundance of Chloroflexi of forest gaps in winter was significantly higher than that of the control. Chloroflexi species hydrolyze polysaccharides, such as cellulose, xylan, and chitin (Fierer et al., 2012), and they generate energy through solar radiation and 3-hydroxypropionate bicycle at different nutritional levels (Klatt et al., 2013), resulting in forest gaps promoting the growth of these bacteria (Tripathi et al., 2016; Yabe et al., 2016). In this study, the main bacteria genera in the three forest gap sizes were the same and these similarities reflected the stability of the soil bacterial community, which is only broken by long-term disturbance (Jin et al., 2019). These results confirmed that forest gaps alter the soil bacterial community composition and alpha diversity.

In this study, the co-occurrence network of the soil bacterial community found that many taxa with high network centrality were the dominant species at the phyla level, such as Acidobacteria, Chloroflexi, Rokubacteria, Bacteroidetes, Proteobacteria, and Firmicutes, indicating that these generalists are adapted to a variety of environments (Jiao et al., 2016). In the co-occurrence network at the species level, the relationship between species was positively correlated, which indicated that the species promoted each other and shared resources, and these results suggested that metabolic cooperation may have played an important role in shaping species co-occurrence (Zelezniak et al., 2015). Specifically, *Lactobacillus_murinus, Clostridium_perfringens_CPE_str_F4969, Lactobacillus_gasseri*, and *Solitalea_koreensis* all played an important role in network centrality, but they had low relative abundances. Importantly, these taxa should not be overlooked because they may have unique characteristics or have an impact on other species, thus affecting the function of the ecosystem (Jousset et al., 2017). Therefore, forest gaps not only have an essential impact on the soil bacterial composition but also on the relationships between individuals.

### Effects of Soil Physiochemical Properties on the Bacterial Community Composition and Diversity

Recent studies have demonstrated that soil is the key driver of the composition and diversity of microbial communities across various environments (Ma et al., 2017; Ren et al., 2018a). Many studies have shown that soil bacterial diversity is affected by TP (Tan et al., 2013), SOM, and pH (Ren et al., 2018b), but these effects were not identified in this study. The disturbance of afforestation disturbs the soil water and nutrient status, which affects the bacterial diversity (Jangid et al., 2011). The variation of SM with time affects the alpha diversity of soil bacteria (Lin et al., 2018). In addition, BD affects water infiltration and plays an important role in maintaining the availability of water (Salazar et al., 2009; Wang et al., 2015), and SM influences soluble nutrient uptake and microbial activity (Gao et al., 2020). In agreement, our study demonstrated that SM and BD had the most significant effect on bacterial alpha diversity and were positively correlated with alpha diversity.

The RDA indicated that MBC mainly affected the soil bacterial community at the phyla level, and the total variance of the core bacterial community at the genera level was mainly affected by AN and C/N ratio. MBC accounts for only a small part of soil TC, but it is closely related to soil microorganisms and directly reflects the changes in the state and function of the microbial community (Li et al., 2018; Singh and Gupta, 2018). The bacterial community is mainly affected by the supply of soil carbon and nitrogen (Liu et al., 2020), and the AN determines the composition of the bacterial community at the genera level. Xue et al. (2017) found that the close relationship between the C/N ratio and bacterial community structure reveals that the variables involved in nitrogen transformation may be key determinants of the bacterial community structure. A lower C/N ratio provides a rich substrate for the growth of bacterial communities, which may lead to larger bacterial communities and higher activity (Wardle et al., 2004). This study demonstrated that soil physiochemical properties were closely related to soil bacterial composition and diversity. In addition, there are additional environmental factors that may affect the composition and diversity of soil bacterial communities, requiring more in-depth research to explore their specific mechanism.

## Conclusion

This study demonstrated that compared to non-gaps, forest gaps improved the soil properties of weeping cypress plantations and increased the soil bacterial community diversity. The composition of dominant groups in the soil bacterial community was similar in winter and summer, and Proteobacteria was the most abundant phylum. The soil bacterial diversity in summer was more sensitive than that in winter and was more easily affected by forest gaps. Soil physicochemical properties significantly affected the composition and diversity of the soil bacterial community. Large forest gaps were beneficial to the accumulation of soil SOC, TN, AN, and SM, thus promoting soil bacterial community diversity and the distribution of dominant communities. In forest management, if the improvement of the soil environment under the conditions of a similar stand is considered, these results showed that the establishment of large forest gaps to improve the plantation will help to alter soil fertility and soil bacterial community structure. Furthermore, biotic or abiotic factors, such as soil enzymes or plant traits, are also key factors affecting the soil bacterial community structure. Thus, future studies should focus on more detailed environmental factors.

## Data Availability Statement

The datasets presented in this study can be found in online repositories. The names of the repository/repositories and accession number(s) can be found at: https://www.ncbi.nlm.nih.gov/, sra/PRJNA796764.

## Author Contributions

QL, YL, and XL conceived of the study and designed the methodology. QL, YL, YX, and YD conducted field sampling. QL and YL performed the laboratory work. QL analyzed the data and wrote the first draft of the manuscript. GC and KZ assisted with revising the draft manuscript. CF and YC provided laboratory resources. All authors approved the final manuscript.

## Funding

This research was supported by the German Government loans for Sichuan Forestry Sustainable Management (Grant No. G1403083) and a Pillar Project of the 12th Five-Year Plan for China (Grant No. 2011BAC09B05).

## Conflict of Interest

The authors declare that the research was conducted in the absence of any commercial or financial relationships that could be construed as a potential conflict of interest.

## Publisher's Note

All claims expressed in this article are solely those of the authors and do not necessarily represent those of their affiliated organizations, or those of the publisher, the editors and the reviewers. Any product that may be evaluated in this article, or claim that may be made by its manufacturer, is not guaranteed or endorsed by the publisher.
