# Supplementary material for: Effects of Forest Gaps on the Structure and Diversity of Soil Bacterial Communities in Weeping Cypress Forest Plantations
Source: Front Microbiol. 2022 May 16;13:882949. doi: 10.3389/fmicb.2022.882949 (PMC9149315; doi:10.3389/fmicb.2022.882949)
Supplement: Supplementary file 1 [file Table_1.docx]

Supplementary Material

**Supplementary Table 1.** Results of two-way ANOVA for the effects of forest gaps (FG), two seasons (TS), and their interactions (FG * TS) on soil physicochemical properties and soil bacterial Alpha diversity indices. (DF: Degrees of freedom).

| Index |  | FG | TS | FG*TS |
| --- | --- | --- | --- | --- |
| pH | F | 2.24 | 3.33 | 9.07 |
|  | *P* | 0.12 | 0.09 | 0.001 |
|  | DF | 3 | 1 | 3 |
| SOC | F | 61.49 | 8.32 | 16.852 |
|  | *P* | < 0.001 | 0.01 | < 0.001 |
|  | DF | 3 | 1 | 3 |
| TN | F | 222.97 | 288.66 | 13.65 |
|  | *P* | < 0.001 | < 0.001 | < 0.001 |
|  | DF | 3 | 1 | 3 |
| C/N | F | 7.67 | 10.58 | 1.59 |
|  | *P* | 0.002 | 0.005 | 0.23 |
|  | DF | 3 | 1 | 3 |
| TP | F | 34.58 | 23.25 | 0.48 |
|  | *P* | < 0.001 | < 0.001 | 0.70 |
|  | DF | 3 | 1 | 3 |
| AP | F | 4.13 | 3.30 | 2.11 |
|  | *P* | 0.02 | 0.09 | 0.14 |
|  | DF | 3 | 1 | 3 |
| AN | F | 4.63 | 6.53 | 2.03 |
|  | *P* | 0.02 | 0.02 | 0.15 |
|  | DF | 3 | 1 | 3 |
| MBC | F | 0.09 | 29.59 | 0.57 |
|  | *P* | 0.97 | < 0.001 | 0.64 |
|  | DF | 3 | 1 | 3 |
| MBN | F | 0.3 | 41.7 | 1.61 |
|  | *P* | 0.82 | < 0.001 | 0.23 |
|  | DF | 3 | 1 | 3 |
| BD | F | 6.98 | 0.01 | 2.11 |
|  | *P* | 0.003 | 0.92 | 0.14 |
|  | DF | 3 | 1 | 3 |
| SM | F | 7.35 | 2.12 | 1.39 |
|  | *P* | 0.003 | 0.1 | 0.28 |
|  | DF | 3 | 1 | 3 |
| Shannon | F | 3.12 | 3.07 | 3.07 |
|  | *P* | 0.06 | 1 | 0.06 |
|  | DF | 3 | 1 | 3 |
| Simpson | F | 11.01 | 6.98 | 11.6 |
|  | *P* | <0.001 | 0.02 | <0.001 |
|  | DF | 3 | 1 | 3 |
| Chao1 | F | 4.42 | 10.53 | 5.74 |
|  | *P* | 0.02 | 0.005 | 0.007 |
|  | DF | 3 | 1 | 3 |
| ACE | F | 5.32 | 1.07 | 7.07 |
|  | *P* | 0.01 | 0.32 | 0.003 |
|  | DF | 3 | 1 | 3 |
