# Supplementary material for: Effects of Forest Gaps on the Structure and Diversity of Soil Bacterial Communities in Weeping Cypress Forest Plantations
Source: Front Microbiol. 2022 May 16;13:882949. doi: 10.3389/fmicb.2022.882949 (PMC9149315; doi:10.3389/fmicb.2022.882949)
Supplement: Supplementary file 2 [file Presentation_1.pdf]

Supplementary Material

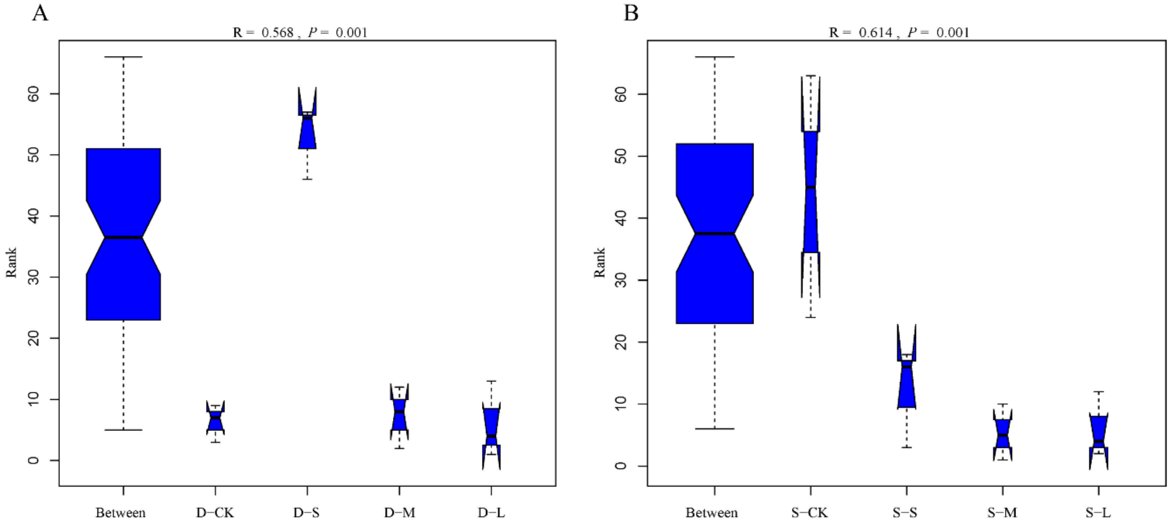

Supplementary Figure 1. The significant differences of the bacterial community composition were evaluated using an analysis of similarities in (A) winter and (B) summer. D-S, represents small gaps in winter; D-M, represents medium gaps in winter; D-L, represents large gaps in winter; D-CK, represents no forest gaps in winter; S-S, represents small gaps in summer; S-M, represents medium gaps in summer; S-L, represents large gaps in summer; and S-CK, represents no forest gaps in summer.

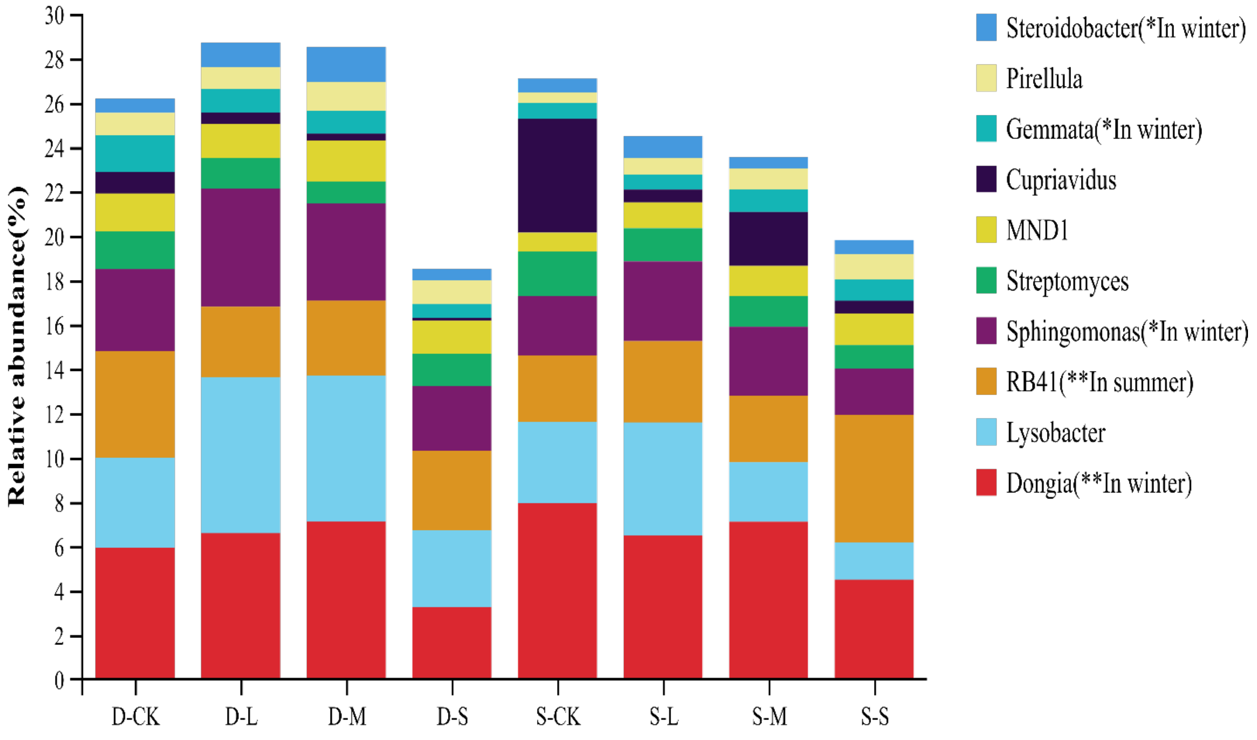

Supplementary Figure 2. Relative abundance of bacteria taxa at the genera level. At the genera level, except for "Other" and "Unclassified", only the taxa with average relative abundance of top ten were shown. *, $P < 0.05$; **, $P < 0.01$. D-S, represents small gaps in winter; D-M, represents medium gaps in winter; D-L, represents large gaps in winter; D-CK, represents no forest gaps in winter; S-S, represents small gaps in summer; S-M, represents medium gaps in summer; S-L, represents large gaps in summer; and S-CK, represents no forest gaps in summer.

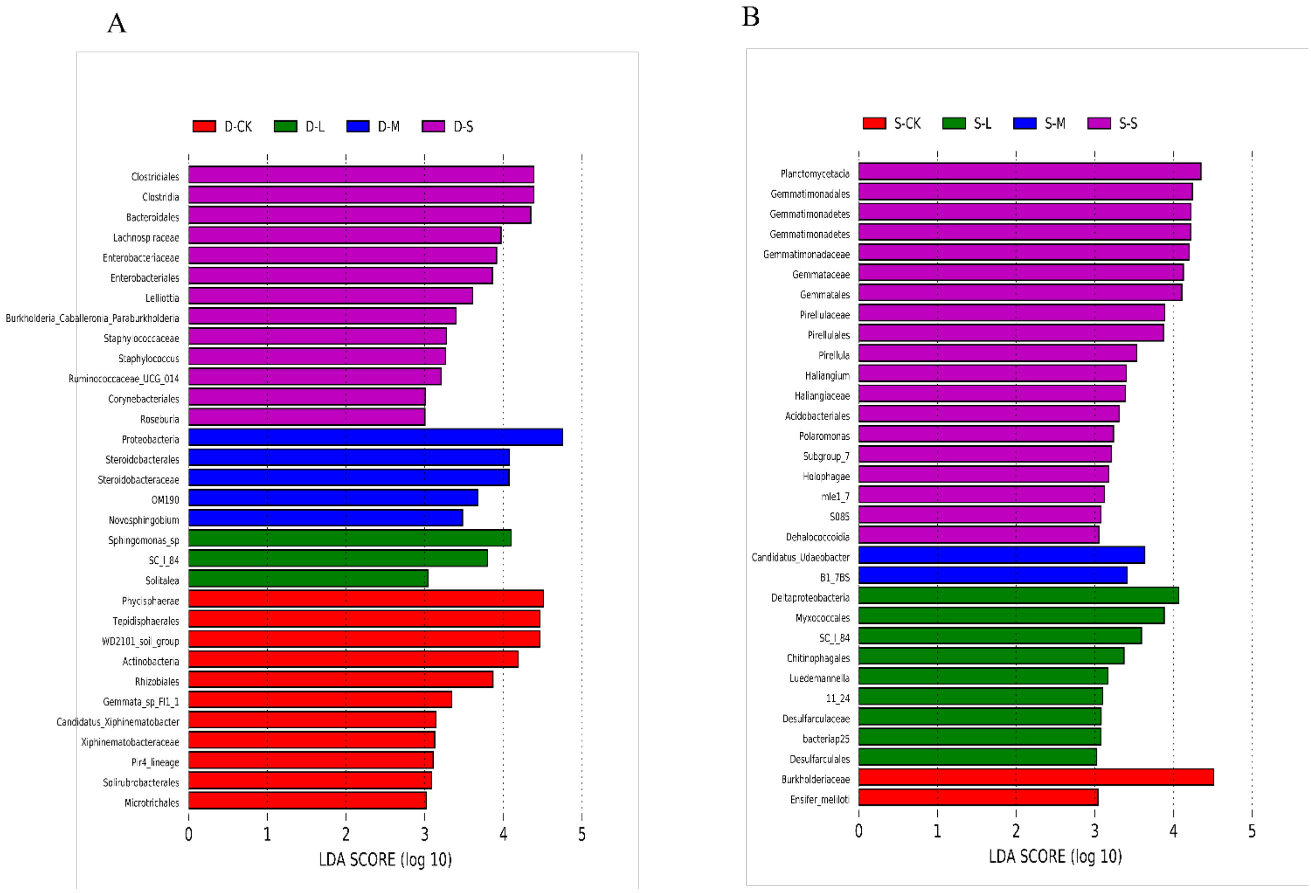

Supplementary Figure 3. Linear Discriminant Analysis (LDA) of the soil bacterial community among forest gap sizes in (A) winter and (B) summer. Only taxa meeting an LDA significance threshold of > 3 are shown. D-S, represents small gaps in winter; D-M, represents medium gaps in winter; D-L, represents large gaps in winter; D-CK, represents no forest gaps in winter; S-S, represents small gaps in summer; S-M, represents medium gaps in summer; S-L, represents large gaps in summer; and S-CK, represents no forest gaps in summer.
